# Dr. Martin’s Suggestion

**Published:** 1919-03

**Authors:** 


### Dr. Martin's Suggestion.

Dr. E. H. Martin, of Hot Springs, known to most of the readers of The Texas Medical Journal as one of the most progressive physicians of the country, was recently asked by the editor of this journal what he thought was the most important question before the medical profession today. His answer, while it may be ahead of the professional thought of the day, should start physicians to thinking. Clearly some change is coming in the relations of physicians to the public—it must come as much for the protection of the doctors themselves as for the good of the people. The building of public and private hospitals throughout the country is seriously effecting the one-man practitioner. Will the movement result in the getting together of the physicians in large firms to handle practically every phase of medical practice and surgery, or will the government, National or State, undertake to care for the sick, as the sick have been cared for in the Army and Navy service? We do not know; no one does.

With the return of the soldiers and sailors to civilian life there is going to be some agitation for the government care of the sick or for medical insurance, or some other more modern form of health preservation than we have had. Many people are coming to regard public health as a public asset, and illness as a national calamity from which the well also suffer. They argue that when one man, one worker, is sick some other worker has to do his work for him; therefore it pays to keep everybody well.

Whether you think Dr. Martin's suggestion is likely to be followed or not, sit down and write what you think about it, or what else may be done instead. The Texas Medical Journal will be glad to have your opinion, even briefly expressed, for "The Medical Letter-Bag," a department it has set aside for short correspondence from physicians.

